# Stated Choice design comparison in a developing country: recall and attribute nonattendance

**DOI:** 10.1186/s13561-014-0025-3

**Published:** 2014-10-24

**Authors:** Richard A Iles, John M Rose

**Affiliations:** 1 Department of Accounting, Finance and Economics, Griffith University, 170 Kessels Road, Brisbane, Australia; 2 Institute of Choice, University of South Australia, Arthur Street, North Sydney, Australia

**Keywords:** Discrete choice experiment, Experimental design, India, Unqualified, Efficient, Orthogonal, Attribute nonattendance

## Abstract

**Background:**

Experimental designs constitute a vital component of all Stated Choice (aka discrete choice experiment) studies. However, there exists limited empirical evaluation of the statistical benefits of Stated Choice (SC) experimental designs that employ non-zero prior estimates in constructing non-orthogonal constrained designs. This paper statistically compares the performance of contrasting SC experimental designs. In so doing, the effect of respondent literacy on patterns of Attribute non-Attendance (ANA) across fractional factorial orthogonal and efficient designs is also evaluated. The study uses a ‘real’ SC design to model consumer choice of primary health care providers in rural north India. A total of 623 respondents were sampled across four villages in Uttar Pradesh, India.

**Methods:**

Comparison of orthogonal and efficient SC experimental designs is based on several measures. Appropriate comparison of each design’s respective efficiency measure is made using D-error results. Standardised Akaike Information Criteria are compared between designs and across recall periods. Comparisons control for stated and inferred ANA. Coefficient and standard error estimates are also compared.

**Results:**

The added complexity of the efficient SC design, theorised elsewhere, is reflected in higher estimated amounts of ANA among illiterate respondents. However, controlling for ANA using stated and inferred methods consistently shows that the efficient design performs statistically better. Modelling SC data from the orthogonal and efficient design shows that model-fit of the efficient design outperform the orthogonal design when using a 14-day recall period. The performance of the orthogonal design, with respect to standardised AIC model-fit, is better when longer recall periods of 30-days, 6-months and 12-months are used.

**Conclusions:**

The effect of the efficient design’s cognitive demand is apparent among literate and illiterate respondents, although, more pronounced among illiterate respondents.

This study empirically confirms that relaxing the orthogonality constraint of SC experimental designs increases the information collected in choice tasks, subject to the accuracy of the non-zero priors in the design and the correct specification of a ‘real’ SC recall period.

## Background

The use of Stated Choice (SC) methods within health economics is widespread. The research focuses of SC studies in this literature are diverse covering a range of perspectives. These include: patient preferences for non-market medical interventions, health professional preferences towards prescribing medicines and treatments, health care priority setting and consumer preferences towards health insurance schemes [1]–[3]. A limited number of SC studies have focused on evaluating the performance of experimental designs, which underpin the use of SC in all studies [4]–[6].

Experimental designs determine the purposeful mixing of choice alternatives’ attributes and their levels. Different statistical properties and constraints governing the mixing of attribute levels are used in many SC studies across the broad field of applied economics [7],[8]. Orthogonal designs often ensure each attribute pair combination appears equal number of times, which (often) results in zero correlation structure between attributes. An alternative group of designs are referred to as efficient. These designs, assuming non-zero prior parameter estimates, mix attribute levels so as to reduce elements of the Asymptotic Variance-Covariance (AVC) matrix.

Despite the established use of efficient designs in several applied economic fields, the health economic literature is firmly centred on applying orthogonal designs [3]. Recent exceptions to this trend exist. Araña et al. [9] in their evaluation of decision rules used a non-orthogonal design with zero-priors following the design study by Carlsson and Martinsson [4]. Hole [10] used the same D-optimality design procedure in SAS with zero set for prior estimates.

In all SC studies, the analyst must select the experimental design prior to going into the field. Whilst in theory efficient designs should perform better, there is little (and mixed) supporting empirical evidence. The work of Louviere et al. [11] indicates that the efficient design results in greater error variance. Bliemer and Rose [7] find that as per theory, efficient designs produce lower standard errors, but not necessarily larger t-ratios as they tend to produce lower scale (higher error variance). Higher scale of a design may be due to the presence of more choice task dominance, which may cause estimation problems.

Despite the above being known, what has not been studied to date are other effects of using different designs. There exists SC literature on design features, which states that designs may influence the outcome or process used in answering the questions shown [12],[13]. Attribute non-Attendance (ANA) is a well-documented survey respondent process aimed at simplifying choice task by reducing the number of attribute level trade-offs [14]–[18]. However, empirical studies to date use a fixed design. This paper seeks to add to the literature by exploring the effect of different experimental design among respondents with low levels of literacy.

This paper statistically compares the effect of respondent literacy on patterns of ANA across fractional factorial orthogonal and efficient designs. Literate and illiterate sub-samples of respondents provide a natural context to compare the cognitive burden of experimental designs and the resulting modelled output [19],[20]. The results of both stated and inferred ANA are presented. The additional consideration of recall bias is included in the analysis, due to the study’s use of a ‘real’ SC scenario.

The current study is based on consumers’ choice of a ‘real’ market good – choice of health care provider to treat fever symptoms in rural north India. Although many SC studies measure preferences for non-market goods, another set of SC studies - termed ‘real’ SC surveys – model choice for market goods [21]–[23]. Respondents’ processing of ‘real’ SC choice tasks is assumed to be aided in part by the association they make with their most recent market transaction across the same alternatives. While the ability to make connections with recent market based decision-making situations is likely to reduce the cognitive load of SC choice tasks, well-established recall problems are expected to exist as well [24],[25].

For the purposes of this research, the terms *orthogonal* and *efficient* are used to refer to fractional factorial orthogonal and efficient designs respectively. Efficient designs relax the orthogonality constraint and allow for some correlation across levels and the use of non-zero priors. The term ‘*efficient’* follows the same use by Scarpa et al. [26] and Bliemer and Rose [7]. The alternate design, *‘orthogonal’,* uses implicit zero prior estimates and maintains orthogonality across attribute levels and also follows the same by Bliemer and Rose [7].

## Methods

###  Attribute nonattendance

Not controlling for ANA in SC choice tasks is accepted as potentially leading to biased willingness-to-pay and welfare estimates [18],[27]. Two methods exist to account for ANA. These are stated and inferred. Stated ANA has respondents state at the time of completing the survey which, if any, attribute level they ignored in making their choice. Inferred uses a latent class model to classify respondents depending on the estimated probability that respondents ignore selected attributes. The literature is inconclusive as to which ANA method is superior [15],[28]. As a result, this study employs both methods.

###  Study context

This study estimates demand for rural allopathic^a^ health care providers in the north Indian state of Uttar Pradesh (UP). This demand is based on the counter-fractural assumption that government rural Bachelor of Medicine and Bachelor of Surgery (MBBS) doctors are always available at their assigned rural Primary or Community Health Centre [29],[30].

Survey respondents for the purposes of the design comparison come from two UP districts covering two of the four economic regions of the state. One district is from the Bundelkhand (southern region) and the other is from the Eastern region. These districts approximately represent the interquartile range of mean per capita income across UP [31]. Three districts were sampled. Respondents from two of the three districts are initially used due to the inclusion of a stated ANA question in these surveys. The sample of all three districts is then used for inferred ANA estimation for comparative purposes. Ethics approval was obtained from Griffith University’s Human Ethics Committee.

###  Design

Consumers’ preferences for outpatient health care services for fever treatment are estimated with data collected through a SC survey. The survey, using two experimental designs, has four alternatives: i) unqualified private sector providers (in Hindi termed *jhola chhaap*)^b^, ii) private MBBS, iii) government MBBS doctors and iv) other category. The attributes of price, recommendations, distance and medicine were selected to construct the design following the collection and analysis of qualitative interview data [32]. Price and recommendation have three attribute levels and the other two attributes have two levels each. The medicine attribute encapsulates two important features of consumer preference for outpatient fever treatment: i) preference for injections, and ii) preference for free medicines from government health centres. For the purposes of compactness of the choice tasks, these two features are considered within the one attribute. The qualitative variables (recommendation, distance and medicine) are effects coded. Table 1 lists the selected alternatives, attributes and levels of the experimental designs (see Additional file 1: Appendix A for example of choice task format). Both designs were generated using the *Ngene* software [33].

**Table 1:** Stated Choice experimental design alternatives, attributes and levels

**Doctor type**	**Price (INR)**^**#**^	**Medicine**	**Distance**	**Recommendation**
**Jhola Chhap**	(30, 60, 90)	Pill,	At Home,	Positive, No Recommendation, Negative
	50, 100, 150	Pill & Injection	In village,	
**Government MBBS**	(1, 10, 20)	Free,	In village,	Positive, No Recommendation, Negative
	1, 25, 50	Extra Charge	5–15 km	
**Private MBBS**	(70, 140, 210)	**Uncertain**	In village,	Positive, No Recommendation, Negative
	100, 200, 300	**Treatment**	5–15 km	
**None of the above**	0	0	0	0

^#^Values in brackets are values used in the pilot study.

Efficient designs, with the use of non-zero priors to maximise the determinant of the Fisher Information matrix, relax the orthogonality constraint to maximise the information gathered from a given set of choice tasks. The parameter estimates from the pilot orthogonal survey are used in the construction of the alternate efficient design. *Ngene* was again used to construct the efficient design.

Among health economics SC studies, the most common measure of experimental design efficiency is D-efficiency [3]. D-efficiency is a measure of a design’s ‘efficiency’ relative to the design with the lowest D-error. There exist three D-error measures related to orthogonal, efficient and Bayesian designs. These measures differ due to the assumed prior parameters used in the experimental design. The two relevant D-error measures for this study are:

$$D_{z}-\mathrm{error}=\det{\left(\Omega_{1}\left(X,0\right)\right)}^{\frac{1}{k}}$$

$$D_{p}-\mathrm{error}=\det{\left(\Omega_{1}\left(X,\tilde{\beta }\right)\right)}^{\frac{1}{k}}$$

In the above D-error measures Ω_1_ is the AVC matrix, *X* is the experimental design, 0 or $\tilde{\beta }$ are the assumed priors and k the number of parameters estimated. As a result, D_z_-error is most appropriately applied to designs that use zero priors and D_p_-error for designs that use non-zero priors. A comparison of D_z_-error and D_p_-errors relevant to this study are presented in Table 2.

**Table 2:** D-error comparisons between orthogonal and efficient designs

**Experimental design specific D-error**	**Priors = 0**	**Priors ≠ 0**
D_z_-error (orthogonal)	0.199	0.222
D_p_-error (efficient)	0.190	0.208

Efficiency measures in the SC experimental design literature often use several ‘error’ measurements. Scarpa et al. [26] argue that efficiency comparison across the orthogonal and efficient designs is not meaningful unless the D-error measurement accounts for the different experimental design assumptions. Table 2 shows that the constraint of orthogonality reduces the ‘efficiency’ of an experimental design, irrespective of whether priors are used. Using the appropriate D-error measurement alone does not provide a balanced comparison. In such a case, the orthogonal design scores 0.199, compared to the efficient score of 0.208. This comparison suggests that the orthogonal design is more efficient. Moreover, within either design the use of zero priors provides D-error scores that are lower. Maintaining the orthogonality constraint the design assuming zero priors is 0.199, compared to the non-zero priors design score of 0.222. The same is also true for the efficient design.

However, holding the priors constant across the two experimental designs shows that the efficient design provides a lower D-error score. When zero-priors are used the efficient design registers a 0.190 D-error, compared to the 0.199. Likewise, when priors are non-zero the efficient design has a D-error of 0.208, compared to 0.222.

The presence of dominant alternatives in choice tasks, while lowering the log-likelihood measures of model fit, limits the amount of information collected from any given choice task. The reduced information on the trade-offs of levels across choice task alternatives increases the risk of biased parameter estimates. Moreover, reliance on log-likelihood based goodness-of-fit measures, such as *ρ*^*2*^ (rho) and Information Criterion, to assess the performance of alternative experimental designs may misrepresent the value of any given design in estimating the preferences of respondents. Table 3 outlines the criteria for measuring the dominance in choice tasks due to design and a count of the dominant choice tasks across all 36 choice tasks in both orthogonal and efficient designs.

**Table 3:** Alternative dominance by design

	**Strict**	**Moderate**	**Weak**
Orthogonal	0	4	9
Efficient	1	2	9
**Definition**	price_a_ < price_b,c_	price_a_ <= price_b,c_	price_a_ <= price_b,c_
	dist_a_ <= dist_b,c_	dist_a_ = lowest	dist_a_ = lowest
	recomm_a_ < recomm_b,c_	recomm_a_ <= recomm_b,c_	recomm_a_ <= recomm_b,c_
	med_a_ = best	med_a_ = best	
	med_b_ = worst	med_b_ = worst	

where _a_ and _b_ represents either unqualified or government MBBS providers and _c_ private MBBS provider.

### Samples

Rural blocks and their corresponding villages were selected in a stratified quasi-random sampling frame. District administrative blocks were randomly selected and from selected blocks, gram panchayats (local level of administration with elected council governing a collection of four to seven villages) were then randomly selected. Relationships with elected village leaders in each of the sample villages were developed during several preliminary visits associated with collecting qualitative data. Village households were randomly selected by enumerators with either the personal or delegated assistance of village leaders. As per ethics approval, no incentives were given to respondents and all respondents were verbally informed as to their right to end their involvement in the survey without penalty. Respondents provided verbal consent before commencing any surveys.

A total of 623 respondents were sampled across four villages in September 2012. A total of 5607 choice tasks were completed. The Bundelkhand sample (district 1) answered 3285 choice tasks from each design and from the Eastern region (district 2) the sample was 2322. Each respondent answered choice tasks from one randomly assigned design. On average the survey was completed in 25 minutes. The majority of this time was spent introducing the survey in reduced form (i.e. 2 alternatives and 2 attributes). Respondents were then progressively stepped through larger choice tasks until the enumerator was satisfied the respondent understood the concept of the survey.

A select set of socio-economic characteristics of the sample answering orthogonal and efficient choice tasks in districts 1 and 2 is presented in Table 4. Respondents of each design were statistically equivalent in respect to their age, gender, religious orientation, mean income and education attainment at the five per cent level of significance, using a two-tail test. The mean household income ranges between Indian rupees (INR) 54,485 and 51,603 across the two designs. Almost half of all respondents self-report themselves as being illiterate. Respondents in these districts attained at least some primary school education (11 and 13 per cent), completed primary school (13 and 14 per cent) or attained some high school education (8 and 8 per cent).

**Table 4:** Descriptive statistics of district 1 and 2 samples

	**Efficient**			**Orthogonal**		
	**Mean/Per cent**	**St. dev**	**Min/Max**	**Mean/Per cent**	**St. dev**	**Min/Max**
Age	40.5	15.2	18/80	39.7	15.6	18/88
Age <30 (%)	34.4			35.3		
Age >55 (%)	22.2			21.2		
Female (%)	47.6			48.1		
Household size	7.27	3.9	1/29	7.37	3.4	2/23
**Religion (%)**						
Hindu	81.0			76.0		
Brahmin	11.3			10.6		
Kshatriya	3.9			4.2		
Vaishya	36.7			32.7		
Sudhra	24.4			23.4		
Tribe	4.5			5.4		
Muslim	19.0			23.4		
**Income**						
Household income p.a*	54485	46901	1000/350000	51603	36653	14000/300000
Personal income p.a*	17580	24439	-/200000	17887	22967	-/154000
**Education (%)**						
Illiterate	46.9			45.2		
< Primary	10.6			12.8		
Primary	12.9			13.8		
< High	14.5			16.0		
High	8.0			7.7		
Intermediate	4.2			3.2		
University	2.6			0.9		
**Sample size**	**311**			**312**		

*Mean figures rounded.

The recall periods of the most recent episode of fever are also consistent across respondents in the two experimental designs. Table 5 outlines the proportion of respondents within each recall period for the pilot study and both experimental designs. Across the progressively more distant recall periods of 14 or less days, 15–30 days and 2–6 months, the proportion of respondents who answered the efficient design ranged between 21 and 27 per cent. The proportion of respondents who answered the orthogonal designs SC surveys ranged between 22 and 23 per cent. By comparison, the recall periods nominated by pilot study respondents is weighted more heavily towards the more distance periods of one to five years.

**Table 5:** Percentage of district 1 and 2 samples, by recall

	**Pilot**	**Efficient**	**Orthogonal**
**Recall**			
≤ 14 days	18.8	27.0	22.1
15-30 days	6.3	23.8	23.4
2-6 months	18.8	21.2	22.4
7-12 months	18.8	14.5	16.7
1-5 years	37.5	13.2	14.7
Sample size	16	311	312

## Results

### Full trade-off modelling

A multinomial logit (MNL) model is used to identify the determinants of consumer choice of health providers in rural north India. The use of a MNL model follows the empirical design comparison of Bliemer and Rose [7]. *Nlogit (version 5)* was used for all modelling. The probability of making a choice is estimated with the following MNL form

(1) $$\mathrm{Prob}\left(y_{i}=\;j\right)\;=\frac{\exp\left(\beta^{'}x_{\mathrm{ij}}\right)}{\sum_{m=1}^{J_{i}}\exp\left(\beta^{'}x_{\mathrm{im}}\right)}$$

where *j* is the number of alternatives, **x**_i_ is a vector of consumer (i.e. decision-maker) characteristics and *i* represents an individual consumer. This model assumes that each choice made is independent from all others (IID assumption).

The parameter estimates of designs, assuming full attribute trade-offs for the samples in districts 1 and 2, are presented in Table 6. The paired parameter estimate and standard error that have a lower standard error and higher parameter estimate from each design are shown in bold text in Table 6. In total, there are only six pairs across the two designs that display this pattern – five from the orthogonal design and one from the efficient. The parameter estimate signs across the designs in Table 6 are generally consistent, however, signs for i) *Distance* (unqualified), ii) *Recommendations* (private MBBS), and iii) *positive Recommendation* (government MBBS) differ. The coefficient signs in the efficient design for i) and ii) are as expected. The third is not.

**Table 6:** Full trade-off with MNL and full recall period

	**Efficient**			**Orthogonal**		
	**Coeff.**	**St. err^**	**t-ratio**	**Coeff.**	**St. err^**	**t-ratio**
**Unqualified –*jhola chhap*– doctor**						
Constant	−0.354	0.007	−1.03	0.234	0.004	1.10
Price	−0.020	<0.001	−13.01	**−0.020**	**<0.001**	−14.44
Distance (in village)	−0.088	0.001	−1.62	0.078	0.001	1.46
base: at home						
Medicine (pill + Inject)	** 0.136**	**0.001**	2.54	0.136	0.001	2.52
base: (pill)						
Recomm. +ve	−0.021	0.001	−0.28	−0.032	0.001	−0.44
base: no recomm.						
Recomm. +ve	−0.135	0.002	−1.54	−0.043	0.002	−0.55
base: no recomm.						
**Private MBBS**						
Price	−0.022	<0.001	−9.22	−0.016	<0.001	−15.30
Distance (5–15 km)	−1.796	0.003	−12.38	−1.206	0.002	−12.65
base: in village						
Recomm. +ve	0.324	0.002	2.61	−0.322	0.002	−2.62
base: no recomm.						
Recomm. –ve	−0.298	0.003	−2.24	0.283	0.002	2.68
base: no recomm.						
**Government MBBS**						
Constant	−1.285	0.007	−3.65	−0.802	0.003	−4.48
Price	−0.012	<0.001	−4.03	−0.011	<0.001	−4.02
Distance (5–15 km)	−1.512	0.001	−24.81	**−1.617**	**0.001**	−29.41
base: in village						
Medicine (extra INR)	−0.321	0.001	−4.30	**−0.462**	**0.001**	−8.70
base: free						
Recomm. +ve	−0.291	0.002	−3.18	0.097	0.001	1.34
base: no recomm.						
Recomm. –ve	−0.095	0.002	−1.16	**−0.113**	**0.002**	−1.47
base: no recomm.						
**None**						
Constant	−5.536	0.007	−14.53	**−5.575**	**0.005**	−20.12
LL	−1951.2			−1849.6		
AIC	3936.4			3733.1		
AIC/n	1.4			1.3		
*ρ*^*2*^	0.318			0.360		
n	2799			2808		

^Standard error divided by the square root of the sample.

**Bold** text highlights the pair of coefficients (higher) and modified standard errors (lower) than the corresponding design.

The model fit statistics indicate that the orthogonal design fits the data better than the corresponding efficient design and data. The Akaike Information Criteria (AIC) for the orthogonal design and data are also lower. This is also true for the standardised AIC. A *ρ*^2^ of 0.36 translates into a standard linear regression-based coefficient of determination measure of approximately 0.7 [34]. The efficient design *ρ*^2^ is 0.318. The *ρ*^2^ measure continued to show that the orthogonal design/data is a better fit, however, this measure is dataset specific and so should not be the basis for comparison.

The parameter t-ratios from the orthogonal design are also consistently higher than those from the efficient design. Twelve t-ratios from the orthogonal design are larger than their corresponding efficient design t-ratios. Of these, nine are statistically significant at the five per cent level.

A subset of the sample data from each design, based on those respondents who reported having at least a mild fever within the previous 14-days, in Table 7, indicates that the efficient experimental design construction better fit the sub-set of sample data. Model estimates are based on three alternatives, due to the lack of choice on the ‘none’ category among respondents reporting fever in the past 14-days. A total of seven pairs of parameter estimates and (modified) standard errors conform to expectations – these are marked in bold in Table 7. All of these are from the efficient design results. The coefficient estimates for i) *Distance* (unqualified), ii) *positive Recommendation* (unqualified), and iii) *positive Recommendation* (government MBBS) differ between the two designs. Those associated with the efficient design are as expected.

**Table 7:** Full trade-off with MNL and ≤ 14 day recall

	**Efficient**			**Orthogonal**		
	**Coeff.**	**St. err^**	**t-ratio**	**Coeff.**	**St. err^**	**t-ratio**
**Unqualified –*jhola chhap*– doctor**						
Constant	0.314	0.010	1.11	0.692	0.012	2.36
Price	**−0.021**	**<0.001**	−7.27	−0.014	<0.001	−5.56
Distance (in village)	−0.126	0.003	−1.30	0.127	0.004	1.26
base: at home						
Medicine (pill + Inject)	**0.369**	**0.004**	3.63	0.140	0.004	1.38
base: (pill)						
Recomm. +ve	**0.260**	**0.005**	1.96	−0.008	0.006	−0.05
base: no recomm.						
Recomm. +ve	−0.065	0.005	−0.45	−0.168	0.006	−1.15
base: no recomm.						
**Government MBBS**						
Price	−0.031	<0.001	−6.24	0.000	<0.001	0.01
Distance (5–15 km)	**−1.323**	**0.004**	−11.83	−1.417	0.004	−13.30
base: in village						
Medicine (extra INR)	−0.682	0.004	−5.68	−0.430	0.004	−4.14
base: free						
Recomm. +ve	**0.346**	**0.005**	2.47	−0.119	0.006	−0.83
base: no recomm.						
Recomm. –ve	**−0.764**	**0.005**	−5.77	−0.188	0.006	−1.27
base: no recomm.						
**Private MBBS**						
Constant	**−2.348**	**0.007**	−12.44	−1.181	0.007	−6.47
LL	−561.6			−482.8		
AIC	1147.7			989.5		
AIC/n	1.5			1.6		
*ρ*^*2*^	0.239			0.228		
n	765			621		

^Standard error divided by the square root of the sample.

**Bold** text highlights the pair of coefficients (higher) and modified standard errors (lower) than the corresponding design.

The standardised AIC scores show that the efficient design and data provide a better fit to the MNL model. The efficient design has a standardised AIC of 1.50 compared to the orthogonal result of 1.60.

**Figure 1:**
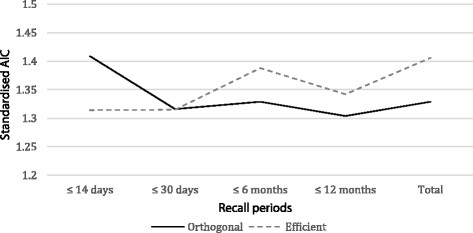
Full trade-off with MNL standardised AIC by recall period.

Continued modelling using progressively longer recall periods and four alternatives shows that the standardised AIC measure of the two designs converged and then the orthogonal design consistently has lower scores. Figure 1 presents a plot of the standardised AIC scores across the recall periods: ≤ 14-days, ≤ 30-days, ≤ 6-months, ≤ 12-months and all recall periods. The sub-set of data using a 30-day or less recall period sees the standardised AIC measures for the efficient and orthogonal designs converge. The same measures for longer recall periods consistently see the orthogonal design and data perform better than the efficient design and data.

### Stated ANA modelling

Following on from the more consistent performance of the full trade-off MNL model of 14-day or less recall period (see Table 7), results from the same model and recall period, controlling for ANA and design dominance, show even lower standard errors and higher coefficients. Table 8 shows these results for each experimental design according to sub-set of choice taks that are defined as i) at least weakly dominant, and ii) the remaining choice tasks with no defined design dominance. In the two ‘no dominance’ group of choice tasks the efficient design has a total of 8 (out of a total of 12) coefficient and standard error pairings conform to theory with lower standard errors and higher coefficients. In contrast, in the ‘weak dominance’ group of choice tasks the orthogonal design has five paired coefficient and standard errors that were jointly higher and lower than the corresponding pair in the efficient design. The standardised AIC scores for both groups of efficient choice tasks are lower.

**Table 8:** Stated ANA with MNL and ≤ 14-day recall

	**Efficient**						**Orthogonal**					
	**Weak dominance**			**No dominance**			**Weak dominance**			**No dominance**		
	**Coeff.**		**St. err^**	**Coeff.**		**St. err^**	**Coeff.**		**St. err^**	**Coeff.**		**St. err^**
**Unqualified –*jhola chhap*– doctor**												
Constant	−0.130		0.023	−0.265		0.009	**0.748**	**	**0.018**	0.301		0.011
Price	−0.007	*	<0.001	**−0.015**	***	**<0.001**	**−0.017**	***	**<0.001**	−0.011	***	<0.001
Distance (in village)	−0.300	*	0.009	−0.117		0.004	0.037		0.008	0.163		0.004
base: at home												
Medicine (pill + Inject)	0.153		0.009	**0.381**	***	**0.004**	−0.072		0.007	0.169		0.004
base: (pill)												
Recomm. +ve	1.350	***	0.021	**0.398**	***	**0.005**	**0.259**		**0.011**	0.199		0.006
base: no recomm.												
Recomm. +ve	−0.831	***	0.015	−0.201		0.006	−0.737	***	0.012	−0.440	**	0.007
base: no recomm.												
**Government MBBS**												
Price	−0.001		0.001	**−0.034**	***	**<0.001**	**−0.017**	**	**<0.001**	−0.005		<0.001
Distance (5–15 km)	−1.660	***	0.011	−1.463	***	0.004	**−1.744**	***	**0.008**	−1.586	***	0.005
base: in village												
Medicine (extra INR)	−1.295	***	0.014	**−0.860**	***	**0.005**	−0.704	***	0.008	−0.540	***	0.005
base: free												
Recomm. +ve	1.448	***	0.019	**0.631**	***	**0.006**	−0.287		0.011	0.057		0.007
base: no recomm.												
Recomm. –ve	−1.131	***	0.016	**−0.943**	***	**0.005**	−0.391	*	0.011	−0.442	**	0.007
base: no recomm.												
**Private MBBS**												
Constant	−1.796	***	0.014	**−2.325**	***	**0.007**	−1.316	***	0.011	−1.213	***	0.007
LL	−199.7			−545.3			−284.1			−458.8		
AIC	423.4			1115			592.3			941.5		
AIC/n	1.2			1.5			1.5			1.6		
*ρ*^*2*^	0.328			0.251			0.283			0.253		
n	359			748			413			608		

**^**Standard error divided by the square root of the sample.

**Bold** text highlights the pair of coefficients (higher) and modified standard errors (lower) than the corresponding design.

* 10%, ** 5%, *** 1% statistical significance.

Controlling for stated ANA helps provide more consistent coefficients. Only one set of coefficients have differing signs in the results summarised in Table 8. The positive Distance (unqualified) coefficient continues in the orthogonal design ‘weakly dominant’ and ‘no dominance’ groups.

A Latent Class (LC) model allows for parameter heterogeneity across individual respondents with the use of discrete latent classes. Individuals are allocated to a class according to a non-parametric marginal probability. Within each class, choice probabilities are generated using a MNL model. Equation ([2]) outlines the probabilistic structure for an individual choice task as the expected value (*E*_*c*_)

(2) $$\mathrm{Prob}\;\left(y_{\mathrm{it}}=\;j\right)\;=E_{c}\left[\frac{\exp\left(\beta_{c}^{'}x_{\mathrm{jit}}\right)}{\sum_{j=1}^{J_{i}}\exp\left(\beta_{c}^{'}x_{\mathrm{jit}}\right)}\right]\text{,}$$

where the subscript *t* denotes individual choice task completed by a given respondent and *c* denotes class. Expanding the expected value over all nominated classes equation ([2]) is re-written as

(3) $$\mathrm{Prob}\;\left(y_{\mathrm{it}}=\;j\right)\;=\sum_{c=1}^{C}\mathrm{Prob}\left[\mathrm{class}=c\right]\left[\frac{\exp\left(\beta_{c}^{'}x_{\mathrm{jit}}\right)}{\sum_{j=1}^{J_{i}}\exp\left(\beta_{c}^{'}x_{\mathrm{jit}}\right)}\right]\text{.}$$

The probability that a respondent is allocated to a given class is represented in equation ([4]) below in a multinomial logit form,

(4) $$\mathrm{Prob}\;\left(\mathrm{class}=c\right)\;=\frac{\exp\left(\theta_{c}\right)}{\sum_{c=1}^{C}\exp\left(\theta_{c}\right)}\text{.}$$

Equation ([4]) includes only fixed constants and no covariates. This reflects the inferred ANA purpose of the LC model. The last *θ*_*c*_ is set at zero. Combining equations ([3]) and ([4]) to reflect the nine choice tasks faced by respondents as part of the current research, the probability of the sequence, conditional on being in class *c* is

(5) $$\mathrm{Prob}\left(y_{i1,\ldots ,}y_{i9}\right)\;=\sum_{c=1}^{C}\frac{\exp\left(\theta_{c}\right)}{\sum_{s=1}^{C}\exp\left(\theta_{c}\right)}\prod_{t=1}^{t=9}\frac{\exp\left(\beta_{c}^{'}x_{\mathrm{jit}}\right)}{\sum_{j=1}^{J}\left(\beta_{c}^{'}x_{\mathrm{jit}}\right)}\text{.}$$

The use of a LC model, being more data intensive, requires a comparison of the designs using a larger sample. Data from a third district in UP is included. This additional data was not included in the previous MNL modelling due to the lack of information on respondents’ stated ANA. The descriptive statistics of the new sample are consistent with those in Tables 4 and 5.

###  Inferred ANA class probabilities

Using inferred methods to test for the presence of ANA among respondents’ choices, a consistent pattern of higher ANA among the efficient design is evident. This is true among illiterate and literate SC survey respondents. Table 9 shows that in a Latent Class MNL model using four classes among illiterate respondents, the probability that no ANA is employed in answering efficient design surveys range from 0.69 to 0.45. This range contrasts with the range of 0.98 to 0.93, using orthogonal designs. Similarly, among literate respondents in Table 10, the range among those answering a set of efficient design choice tasks is 0.89 to 0.84, while for those using an orthogonal design the range is 0.99 to 0.93. The higher ANA probabilities among illiterate respondents, and illiterate respondents answering efficient designs, supports a priori expectations that the greater cognitive demands for trading-off attributes and their levels in efficient designs induces higher levels of ANA among lower literacy respondents.

**Table 9:** Latent class MNL class probabilities for illiterate respondents from districts 1, 2 and 3 by recall period

			**Efficient**				**Orthogonal**			
**Recall #**	**Recall period**		**No fixed**	**Med. fixed**	**Recom fixed**	**Dist. fixed**	**No fixed**	**Med. fixed**	**Recom fixed**	**Dist. fixed**
1	≤ 14 days	Prob.	0.69	0.31	<0.01	<0.01	0.93	0.06	0.01	n/a
		t-ratio	9.60	4.40	<0.01	<0.01	39.45	2.80	0.84	n/a
2	≤ 30 days	Prob.	0.65	0.34	<0.01	0.01	0.96	0.04	<0.01	<0.01
		t-ratio	9.77	6.10	0.89	0.30	23.32	2.34	<0.01	<0.01
3	≤ 6 months	Prob.	0.45	0.47	0.03	0.04	0.99	0.01	<0.01	<0.01
		t-ratio	16.61	15.96	4.77	2.69	48.00	0.76	<0.01	<0.01
4	≤ 12 months	Prob.	0.67	0.31	0.01	0.01	0.98	0.02	<0.01	n/a
		t-ratio	15.30	7.50	1.56	1.16	57.79	1.96	<0.01	n/a
5	Total	Prob.	0.60	0.37	0.01	0.03	n/a	n/a	n/a	n/a
		t-ratio	13.46	8.92	2.58	3.64	n/a	n/a	n/a	n/a

n/a denotes parameters were not fixed due to non-convergence.

**Table 10:** Latent class MNL class probabilities for literate respondents from districts 1, 2 and 3 by recall period

			**Efficient**				**Orthogonal**			
**Recall #**	**Recall period**		**No fixed**	**Med. fixed**	**Recom fixed**	**Dist. fixed**	**No fixed**	**Med. fixed**	**Recom fixed**	**Dist. fixed**
1	≤ 14 days	Prob.	0.86	0.14	<0.01	<0.01	0.96	0.04	<0.01	n/a
		t-ratio	16.88	2.75	<0.01	<0.01	36.95	1.62	<0.01	n/a
2	≤ 30 days	Prob.	0.84	0.16	<0.01	<0.01	0.95	0.06	<0.01	n/a
		t-ratio	19.03	4.31	<0.01	<0.01	51.89	3.02	<0.01	n/a
3	≤ 6 months	Prob.	0.87	0.12	0.01	<0.01	0.81	0.19	0.001	<0.01
		t-ratio	24.00	3.23	2.81	<0.01	13.74	3.40	0.09	<0.01
4	≤ 12 months	Prob.	0.89	0.09	0.01	0.01	0.95	0.05	0.002	n/a
		t-ratio	21.93	2.27	2.85	0.86	53.27	2.98	0.62	n/a
5	Total	Prob.	0.88	0.10	0.01	0.01	0.17	0.82	<0.01	0.02
		t-ratio	28.75	3.53	2.95	1.61	6.35	26.43	0	0.94

n/a denotes parameters were not fixed due to non-convergence.

The experimental design attribute ‘medicine’ is consistently the second most likely attribute to be ignored by respondents. This is the case for both designs, although at different probability levels. The probability that the ‘recommendation’ and ‘distance’ attributes are ignored by respondents, in both designs, generally have low t-ratios and probabilities under five per cent. For efficient design illiterate respondents, the medicine ANA ranged between 0.47 and 0.31. This range is lower for efficient design among literate respondents – 0.16 to 0.09. No noticeable difference is evident across literacy groups in answering orthogonal design choice tasks where the ranges are: 0.06 to 0.01 for illiterate respondents and 0.18 to 0.04 for literate. The one exception is with the full recall period with the orthogonal design, using literate respondents. In this scenario the ANA for medicine in the orthogonal design has a probability of 0.82.

## Discussion

SC experimental design theory indicates that efficient designs should produce parameter output with lower standard errors and higher coefficients. The AVC provides the link between the relative efficiency of an experimental design and the data driven standard error of a given parameter. A lower appropriate D-error measure of a design is expected to translate into a lower standard error of a given coefficient. The results of this study indicate that the recall period does impact on experimental design model fit. In the base case of full recall and assuming full trade-off, only one efficient design parameter conforms to theory with a lower standard error and higher coefficient. Modelling using a 14-day or less recall sub-set of data provides seven efficient design parameters conforming to design theory.

The selection of recall period has a strong effect on standardised AIC measures of experimental design fit to the respective datasets. The datasets defined by respondents with a 14-day or less recall period consistently show that the efficient design had a better model fit. This effect is evident when: i) full trade-offs are assumed, ii) stated ANA is incorporated into MNL models and iii) inferred ANA is modelled using a LC model (results not shown in paper). The evidence of this pattern under differing attribute trade-off assumptions suggests that the benefits of greater information collected through efficient designs outweighs the potential noise created by inconsistent respondent choices due to cognitively more complex efficient designs.

Controlling for design induced alternative dominance indicates that the estimated results from choice tasks that contain fewer dominant alternatives provide more consistently paired coefficients and standard errors. Among the ‘no dominance’ group of choice tasks the efficient design results continue to provide higher coefficients and lower standard errors. The opposite is true of choice tasks in the ‘weakly dominant’ group. Of the nine choice task from each design defined as at least ‘weakly dominant’, three orthogonal designs induced estimated probabilities for the dominant alternative at ≥ 0.95, while the corresponding number of efficient design choice tasks is five. The lower number of dominant choice task, defined by design and the data, is thought to help produce higher coefficients and lower standard errors.

However, the repeated opposing coefficient signs between the two designs, across several parameters and when controlling for ANA and recall bias, is unexpected. Controlling for non-trading across choice task attributes through stated ANA reduced the number of opposing coefficient signs, however, the *Distance* parameter for the unqualified – *jhola chhaap* - provider remained negative for the efficient design and positive for the orthogonal. The increase in distance travelled to consult a health care provider is expected to reduce the likelihood of consulting that provider, all other things being equal. The positive *Distance* coefficient in the orthogonal design maybe a result of uncontrolled heuristics.

The higher estimated probabilities in the LC model among the orthogonal design, associated with respondents trading across all attributes, indicates that respondents are less likely to employ choice task heuristics. These higher probabilities, relative to efficient design choice tasks in Tables 9 and 10, are evident among literate and illiterate respondents. The interpretation of higher estimated probabilities for ‘no fixed’ attributes at zero, corresponding to reduced likelihood of employing heuristics, is supported by a relatively higher proportion of literate respondents grouped in the ‘no fixed’ category. The lower probability of respondents simplifying orthogonal design choice tasks indicates that these choice tasks are on average less cognitively demanding and less likely to induce respondent choice inconsistencies.

The use of a MNL model as the basis for model fit design comparisons is a limiting factor of this analysis. While MNL output is generally robust against significant biases, the IID assumption is strong and may have an unaccounted affect on model fit. Moreover, the effects of choice task blocking is also not controlled in the study.

## Conclusions

The cognitive demands of efficient designs have a real impact on the likelihood of respondents’ employing choice task heuristics. The effect of the perceived cognitive demand of the efficient design is apparent among literate and illiterate respondents, however, it is more pronounced among illiterate respondents. Accounting for ANA when modelling choice data further enhanced the comparative statistical performance of the efficient design.

The use of an appropriate D-error measure provides an important insight into the performance of different experimental designs. However, as speculated in the literature, the role of alternative dominant choice tasks, either by design and/or ANA, also affects commonly used goodness-of-fit measures. As such care should be taken in the comparative evaluation of alternative experimental designs.

This study empirically confirms that relaxing the orthogonality constraint of SC experimental designs increases the reliable information collected in choice tasks, subject to the correct specification of a ‘real’ SC recall period. As model parameter estimates diverge from the non-zero priors used in the efficient design construction, the relative statistical performance of the orthogonal design improves and outperforms the efficient design. Although not presented in this study, the importance of convergence between non-zero priors and model parameter estimates suggests that SC Bayesian experimental designs would better manage potential non-zero prior uncertainty.

###  Endnotes

^a^Allopathic health care in India is based primarily on the use of pharmaceutical goods to treat symptoms. This is in contrast to traditional Indian systems of medicine.

^b^A range of terms are used to define unqualified allopathic health care providers in India, however, the term *jhola chhap* is widely used among rural north Indian settings and is also acknowledged in legal settings (see: [32]).

## Competing interests

The authors declare that they have no competing interests.

## Authors’ contributions

RI conceived of the study, participated in the experimental design, managed the collection of data, carried out data manipulation and statistical analysis and drafted the manuscript. JR participated in the experimental design and assisted in framing the experimental design comparison. Both authors read and approved the final manuscript.

## Additional file

## Supplementary Material

Additional file 1: Appendix A (presentation of SC choice task in English). Click here for file
