# Supplementary material for: Stated Choice design comparison in a developing country: recall and attribute nonattendance
Source: Health Econ Rev. 2014 Oct 24;4:25. doi: 10.1186/s13561-014-0025-3 (PMC4209457; doi:10.1186/s13561-014-0025-3)
Supplement: Additional file 1: — Appendix A (presentation of SC choice task in English). [file s13561-014-0025-3-S1.docx]

# Additional files

### Additional file 1 – Appendix A (presentation of SC choice task in English)
